# Egg-Derived Tri-Peptide IRW Exerts Antihypertensive Effects in Spontaneously Hypertensive Rats

**DOI:** 10.1371/journal.pone.0082829

**Published:** 2013-11-29

**Authors:** Kaustav Majumder, Subhadeep Chakrabarti, Jude S. Morton, Sareh Panahi, Susan Kaufman, Sandra T. Davidge, Jianping Wu

**Affiliations:** 1 Department of Agricultural, Food and Nutritional Science, University of Alberta, Edmonton, Alberta, Canada; 2 Department of Obstetrics and Gynecology, University of Alberta, Edmonton, Alberta, Canada; 3 Women and Children’s Health Research Institute, University of Alberta, Edmonton, Alberta, Canada; 4 Department of Physiology, University of Alberta, Edmonton, Alberta, Canada; 5 Cardiovascular Research Centre, University of Alberta, Edmonton, Alberta, Canada; INSERM, France

## Abstract

**Background:**

There is a growing interest in using functional food components as therapy for cardiovascular diseases such as hypertension. We have previously characterized a tri-peptide IRW (Ile-Arg-Trp) from egg white protein ovotransferrin; this peptide showed anti-inflammatory, anti-oxidant and angiotensin converting enzyme (ACE) inhibitor properties *in* *vitro*. Given the pathogenic roles played by angiotensin, oxidative stress and inflammation in the spontaneously hypertensive rat (SHR), we tested the therapeutic potential of IRW in this well-established model of hypertension.

**Methods and Results:**

16–17 week old male SHRs were orally administered IRW at either a low dose (3 mg/Kg BW) or a high dose (15 mg/Kg BW) daily for 18 days. Blood pressure (BP) and heart rate were measured by telemetry. Animals were sacrificed at the end of the treatment for vascular function studies and measuring markers of inflammation. IRW treatment attenuated mean BP by ~10 mmHg and ~40 mmHg at the low- and high-dose groups respectively compared to untreated SHRs. Heart rate was not affected. Reduction in BP was accompanied by the restoration of diurnal variations in BP, preservation of nitric oxide dependent vasorelaxation, as well as reduction of plasma angiotensin II, other inflammatory markers and tissue fibrosis.

**Conclusion:**

Our results demonstrate anti-hypertensive effects of IRW *in* *vivo* likely mediated through ACE inhibition, endothelial nitric oxide synthase and anti-inflammatory properties.

## Introduction

Cardiovascular diseases (CVDs) are the single greatest cause of morbidity and mortality worldwide [1]. Hypertension, the persistent increase of blood pressure (BP) at or above 140/90 mmHg, is a major predisposing factor for CVDs [2]. Uncontrolled hypertension leads to widespread chronic damage to the vasculature and contributes to myocardial infarctions, cerebrovascular insufficiency and chronic renal disease [3,4]. The aetiology of hypertension is complex, although important roles for the renin-angiotensin system (RAS), oxidative stress and inflammation have been identified [5-7]. Different therapies exist, yet many patients still have poorly controlled hypertension and remain at increased risk for its complications [8-10]. In addition, use of anti-hypertensive drugs is associated with significant adverse effects [11]. Nutraceuticals or food derived therapies have been explored for many disease conditions as safer alternatives to traditional pharmacological agents [12,13]. Given the significance of hypertension to the global burden of CVDs, there is increasing interest in developing alternative strategies for the management of hypertension.

In addition to their well-known nutritional role, eggs are a rich source of numerous bioactive proteins and peptides with anti-oxidant, anti-microbial and anti-inflammatory properties which may have potential applications as nutraceuticals and/or functional foods [14-16]. We have previously identified a tri-peptide IRW (Ile-Arg-Trp) from enzymatic digestion of the egg white protein ovotransferrin with demonstrated angiotensin converting enzyme (ACE) inhibitory activity *in vitro* [17]. As uncontrolled RAS activity contributes to the pathogenesis of hypertension, this peptide could be of potential therapeutic interest. Recent work from our group has further characterized its anti-oxidant and anti-inflammatory effects on cultured endothelial cells, a site of inflammatory changes that lead to atherosclerosis and consequently, CVDs [18,19]. However, the *in vivo* cardiovascular effects and oral bioavailability of IRW remain unknown, precluding its further development as a viable therapeutic option.

The spontaneously hypertensive rat (SHR) is a well-characterized rodent model for hypertension. SHRs develop persistent hypertension at an early age (~12-14 weeks) and remain hypertensive throughout their lives [20,21]. These animals show increased RAS activity together with increased oxidative stress in the vasculature and a pronounced pro-inflammatory phenotype [5,22-24]. Thus, SHRs have been widely used to study the pathophysiology of hypertension [22,24–26,26]. Various food derived peptides with known ACE inhibitory properties *in vitro* have been used to test for *in vivo* antihypertensive effects in SHR with varying degrees of success [27-29]. Given the anti-oxidant, anti-inflammatory and ACE inhibitory properties of IRW, we used SHRs to study its efficacy as a novel anti-hypertensive agent and the potential mechanisms of its actions.

## Materials and Methods

### Animal model and ethics statement

Fourteen to fifteen week old male SHRs (290.0±10.5 g) were obtained from Charles River (Senneville, QC, Canada). These animals were kept at the University of Alberta animal facility for a week for acclimatization. Rats were exposed to a 12:12 hour, light:dark cycle, in a humidity and temperature-controlled (23°C) environment. All rats were given standard rat chow (0.3% NaCl) and water *ad libitum*. The experimental procedures were approved by the University of Alberta Animal Welfare Committee (Protocol # 611/09/10/D) in accordance with the guidelines issued by the Canada Council on Animal Care and also adhered to the Guide for the Care and Use of Laboratory Animals published by the United States National Institutes of Health.

### Experimental Design

Fifteen to sixteen week old animals were surgically implanted with telemetry transmitters for blood pressure monitoring. A one week period of recovery was allowed following surgery. The animals were then randomly assigned to three treatment groups (n=6): untreated (control), low dose IRW (3 mg/Kg BW) and high dose IRW (15 mg/Kg BW). The doses were selected based on previously published *in vivo* studies on bioactive peptides [27,28]. IRW was dissolved in 20 mL of Ensure (Abbott Nutrition, QC, Canada) and administered once per day for 18 days. Untreated animals were given Ensure alone. BP was recorded for a 24 h period (10 sec of every 1 min) on days 0 (baseline), 3, 6, 9, 12, 15 and 18 under the conditions described above. At the end of the recording period, the animals were sacrificed by decapitation; the blood was collected in EDTA coated tubes (BD vacutainer, NJ, USA), tissues were collected for further analysis and the mesenteric arteries were isolated for vascular function studies. 

### Anesthesia and surgical procedure

A telemetry transmitter was implanted to measure the blood pressure (BP) and heart rate (HR) as follows. Anesthesia was induced using O_2_ and 3% isoflurane, and was maintained throughout the surgical procedure by the administration of O_2_ and 1.5-2% isoflurane. During surgery, body temperature was maintained at 37°C (Homethermic Blanket, Harvard Apparatus, Canada). Animals were then chronically instrumented with a pressure transmitter (PA-C40; Data Sciences International, Minneapolis, MN, USA) according to the manufacturer’s manual. When the animal had reached a surgical plane, an approximately 3-4 cm vertical incision was made on the left side of the spine just above the hip area. Then using Metzanbaum scissors a small pocket was created just under the skin, large enough to fit the transmitter. The transmitter was placed in the pocket and anchored with 4/0 silk suture. The left femoral artery was exposed by an approximate 2 cm long incision on the rat’s left groin area and the cannula (polyethylene, 0.58 mm ID, 0.97 mm OD) of the telemetry probe was then inserted into the femoral artery and advanced up to the aorta. The catheter was then secured at the point of entry to the vessel by using a 4/0 silk suture. All procedures were done under a Zeiss dissecting microscope (Carl Zeiss, Toronto, ON, Canada). After surgery, the rats were caged individually and allowed to recover for one week. During the week of recovery, animals were fed with 50 mL of Ensure (Abbott Nutrition, QC, Canada) to regain post-operative weight, along with rodent chow and water *ad libitum* [30]. For pain management, the rats received one dose (0.05 mg/kg BW) of buprenorphine (0.3 mg/mL) (Animal Resources Centre, McGill University, Montreal, QC, Canada) just after the surgery; this was repeated during the next 2-3 days to a maximum of twice a day, based on the condition of the individual animal.

### Telemetry recording and signal processing

Chronic measurement of mean arterial pressure (MAP) and HR of the animals were performed in a quiet room with minimal electrical interference. Each individual rat cage was placed on top of a receiver (Model RPC-1, ADI instruments, CO, USA) and the signals were recorded through a pressure output adaptor (Model R11CPA, ADI instruments) for measurement of various cardiovascular parameters. Using the data acquisition software LabChart version 7.3 (ADI instruments), the experimental data were recorded continuously in real time. An atmospheric-pressure monitor (Model APR-1, ADI instruments) was also installed to normalize the pressure values received from the transmitters; this provided the actual BP values irrespective of changes in atmospheric pressure. From the MAP signal, systolic blood pressure (SBP) and diastolic blood pressure (DBP) were extracted and HR was calculated between two successive points and expressed in beats per minute (bpm).

### Vascular function

Second order branches of the mesenteric artery were carefully isolated, cleaned of all surrounding adipose and connective tissues and placed in ice-cold HEPES-PSS (in mmol/L: NaCl 142, KCl 4.7, MgSO_4_ 1.17, CaCl_2_ 4.7, K_2_PO_4_ 1.18, HEPES 10 and glucose 5.5; pH 7.4). Arteries with internal diameters ranging 150-250 µm and ~2 mm in length were mounted on two 40 µm tungsten wires (Fine Wire Company, California, USA) and attached to a wire-myograph (DMT, Copenhagen, Denmark) to allow isometric tension recordings. Vessels were normalized through a series of stepwise increases in diameter to determine their optimal resting tension, set to 0.8 x IC100 (the internal circumference (IC) equivalent to 100 mmHg). Following a 30 min equilibration, mesenteric arteries were exposed to a single dose of phenylephrine (PE; 10 µmol/L; Sigma Aldrich, Oakville, Canada) twice, followed by a single dose of methacholine (MCh; 3 µmol/L; Sigma) to assess the functional integrity of the endothelium and smooth muscle. A cumulative concentration response curve to PE (10^-8^ to 10^-4^ mol/L) was performed to determine constrictor responses. To assess endothelial-dependent relaxation, a cumulative concentration response curve to MCh (10^-10^ to 10^-4^ mol/L) was performed. To study the role of nitric oxide (NO) in MCh-dependent relaxation, vessels were studied in presence or absence of the nitric oxide synthase (NOS) inhibitor N_ώ_-nitro-L-arginine methyl ester (L-NAME, 100 µmol/L, Sigma). After L-NAME incubation, the vessels were pre-constricted (80% of maximum) using PE until it reached a plateau response. Cumulative doses of MCh were then added to the bath to assess vascular relaxation. At the end of the experiment, the vessels were exposed to high K^+^ buffer to confirm viability.

### Plasma biomarker analysis

After animal sacrifice, blood samples were collected and kept on ice. The samples were centrifuged (10,000xg for 20 min at 4°C) and the plasma was stored at -80°C until analysis. Both angiotensin II (Ang II) and bradykinin were quantified by respective ELISA kits (Ang II ELISA, Cayman Chemical, Ann Arbor, MI, USA; Bradykinin ELISA, Phoenix Pharmaceuticals, Burlingame, CA, USA) as per the manufactures’ instructions. Commercially available rat cytokine ELISA strips (Signosis Sunnyvale, CA) were used for estimation of cytokines/chemokines in the plasma samples [31]. 

### Endothelial cell culture

Human umbilical vein endothelial cells (HUVECs) were isolated from human umbilical cords as previously described [32], [33][34] . Following collection of umbilical cords, the umbilical vein was first flushed with PBS to remove blood clots and then HUVECs were isolated out using a type 1 collagenase containing buffer. The cells were grown in a humidified atmosphere at 37°C with 5% CO2/95% air in M199 medium with phenol red supplemented by 20% FBS as well as L-Glutamine (Gibco/ Invitrogen, Carlsbad, CA), Penicillin-Streptomycin (Life Technologies, Carlsbad, CA) and 1% endothelial cell growth supplement (ECGS, from VWR International, West Chester, PA). We have previously confirmed the endothelial nature of these cells by staining for the endothelium-specific marker, von Willebrand’s factor (vWF)22. The protocol was approved by the University of Alberta Ethics Committee and the investigation also conformed to the principles outlined in the Declaration of Helsinki and also Title 45, US Code of Federal Regulations, Part 46, Protection of Human Subjects, Revised November 13, 2001, effective December 13, 2001. All subjects provided written informed consent before inclusion into this study. Second passage HUVECS were grown to confluence and treated with 10% SHR plasma (untreated or high dose IRW treated) for 4 hours. Cell lysates were prepared and used for western blotting to measure leukocyte adhesion molecule expression.

### Western blotting

Effect of the IRW treatment on vascular protein expression was determined using western blotting. Frozen (-80°C) aortas and mesenteric arteries from the SHR animals were thawed on ice and homogenized in a protein extraction buffer (20 mmol/L Tris, 5 mmol/L EDTA, 10 mmol/L Na_4_P_2_O_7_, 100 mmol/L sodium fluoride and 1% NP-40) containing 1% (v/v) protease inhibitor cocktail (Sigma). The homogenate was centrifuged at 10,000xg for 10 min at 4°C. Protein concentration in the supernatants was determined by bicinchoninic acid (BCA) assay, using bovine serum albumin as a standard. Samples were stored at -80°C until western blotting. 

Bands for intercellular cell adhesion molecule-1/ICAM-1 (mouse monoclonal antibody, Santa Cruz Biotechnologies, Santa Cruz, CA, USA), vascular cell adhesion molecule-1/VCAM-1 (rabbit polyclonal antibody, Santa Cruz Biotechnologies) and endothelial nitric oxide synthase/ eNOS (mouse monoclonal antibody, BD Biosciences, San Jose, CA, USA) were normalized to ß-actin (rabbit polyclonal antibody, Abcam Inc., Toronto, ON, Canada) or α-tubulin (rabbit polyclonal antibody, Abcam Inc., Toronto, ON, Canada) and expressed as fold change compared to untreated samples run on the same gel. Anti- ß-actin was used at 0.5 µg/mL, anti α-tubulin was used at 0.4 µg/mL, while eNOS, ICAM-1 and VCAM-1 antibodies were used at 1 µg/mL. Goat-anti-rabbit and Donkey-anti-mouse conjugated secondary antibodies (Li-Cor Biosciences, Linclon, NB, USA) were used to visualize the bands in a Li-Cor Odyssey BioImager and quantified by densitometry with corresponding software (Odyssey V3.0, Li-cor Biosciences). 

### Immunofluroscence

Kidney and aorta specimens were embedded in Tissue-Tek O.C.T Compound (Sakura Finetek Europe, Zoeterwoude, Netherlands) and frozen immediately in liquid nitrogen for subsequent analysis. 10 µm tissue sections were prepared, mounted on glass slides at -20 °C and stored at -80 °C. On the day of the experiments, tissue sections were first fixed in acetone and incubated with blocking buffer (1% bovine serum albumin in phosphate-buffer saline) for 30 min. The sections were then immunostained for 2 h at room temperature with rabbit polyclonal antibodies against nitrotyrosine (Dilution 1:200; Chemicon, Temecula, CA, USA) or type I collagen (Dilution 1:200; Novus Biologicals, Littleton, CO, USA). Incubation with the secondary antibody (Dilution 1: 150; Alexa Fluor 546 (red), Invitrogen, Burlington, ON, Canada) was done for 30 min in the dark. Glass cover-slips were mounted with a Vectashield H-1200 Mounting Kit, containing nuclear stain, DAPI (Vector Laboratories, Burlington, ON, Canada), and immediately visualised under an Olympus IX81 fluorescence microscope (Olympus, Tokyo, Japan). Images were obtained using SlideBook imaging software (Olympus) and presented at 100x magnification. A control image with secondary antibody alone was used to detect any nonspecific binding (data not shown). The images were then quantified by subtracting the background fluorescence in the control image, so only the fluorescence from specific immunostaining was visible.

### Statistics

**Figure 1:**
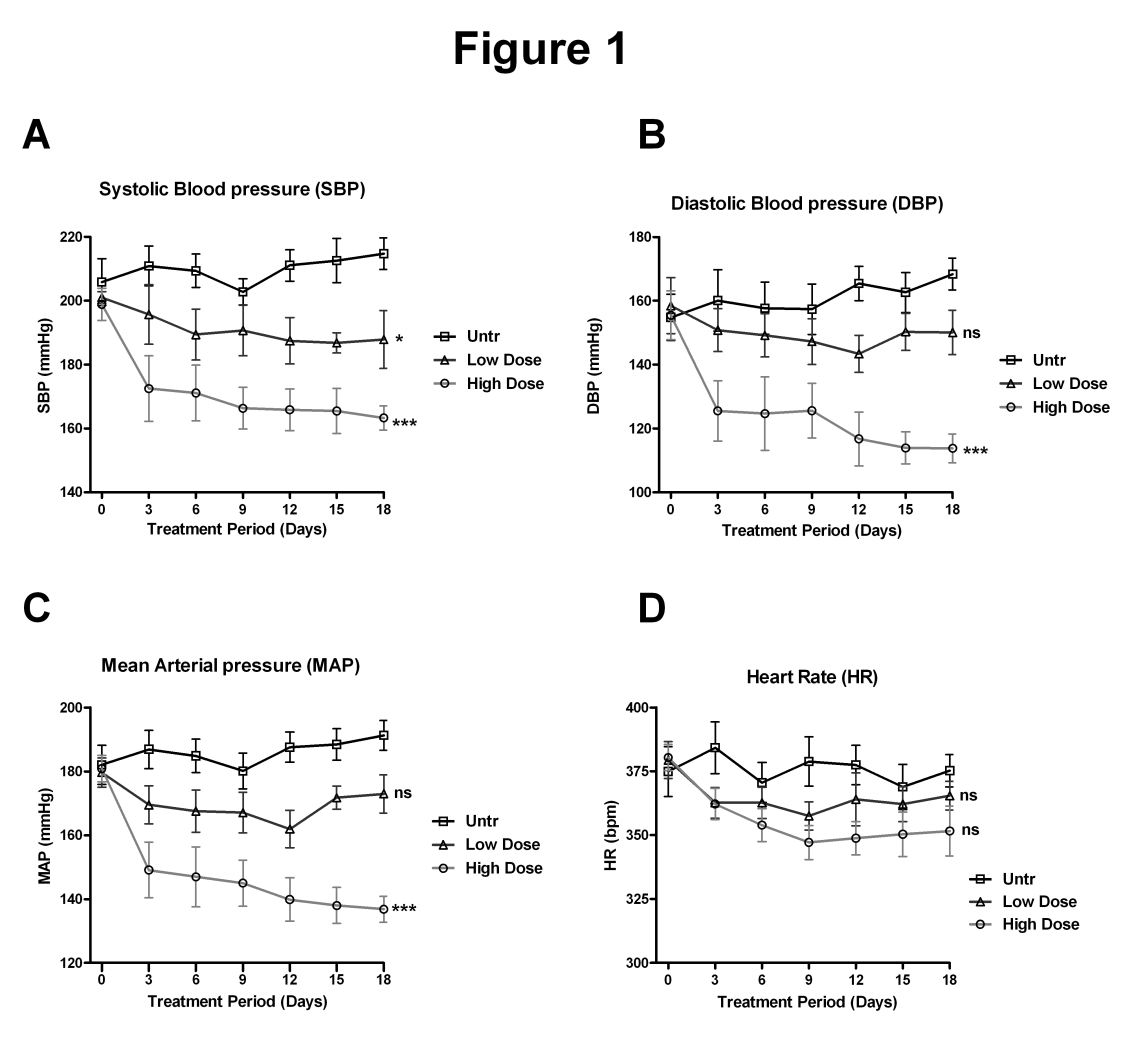
IRW administration lowers BP in SHRs. (A, B and C) SBP, DBP and MAP (mmHg) values from SHRs left untreated (Untr) or treated with a low dose (3mg/Kg BW) or high dose (15mg/Kg BW) of IRW over period of 18 days. BP values for each time point represent the mean BP recorded over a 24 hr period. (D) Heart rate (bpm) of SHRs in the 3 treatment groups over a period of 18 days. Data represented as mean ± SEM from n=6 animals per treatment group. * and *** indicate P<0.05 and P<0.001 respectively, as compared to the untreated group. ‘ns’ indicates not significant compared to the untreated group.

**Figure 2:**
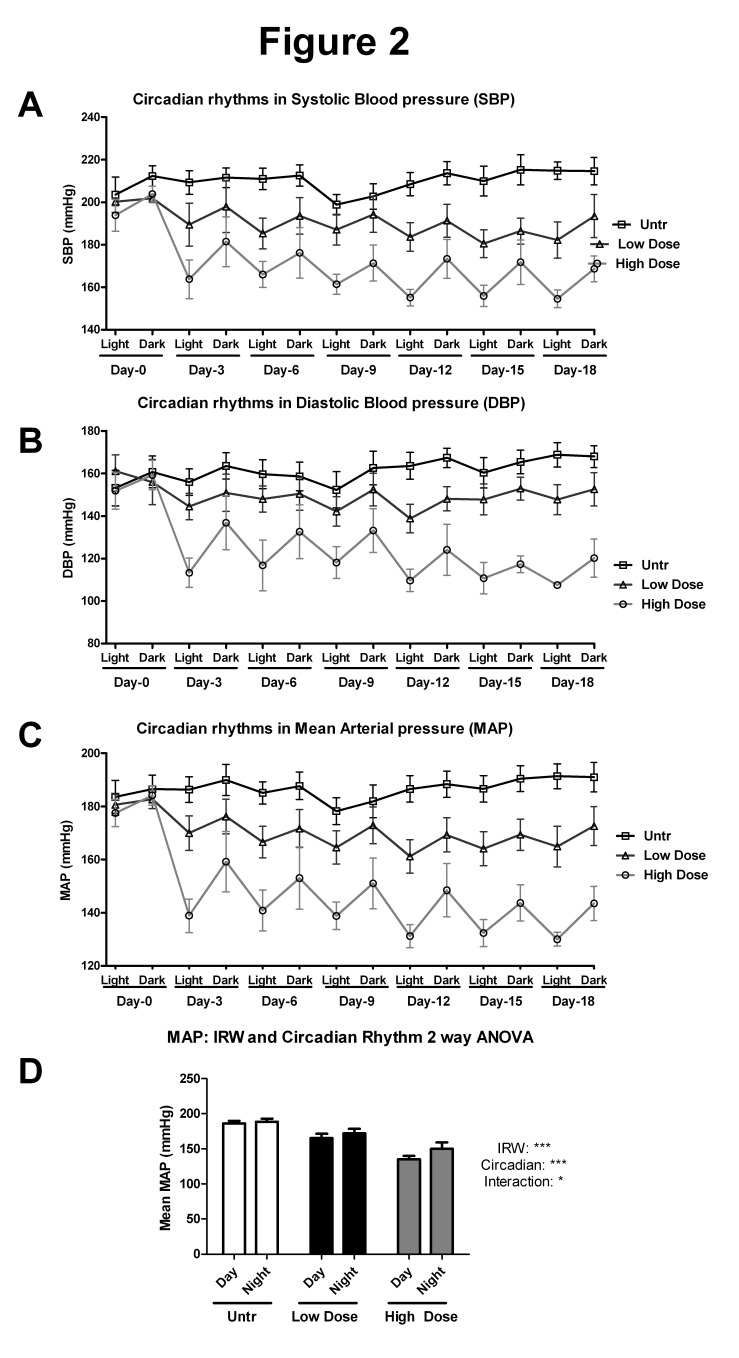
IRW treatment restores the circadian rhythms of BP in SHRs. (A, B and C) SBP, DBP and MAP (mmHg) values from SHRs left untreated or treated with a low dose (3mg/Kg BW) or high dose (15mg/Kg BW) of IRW were recorded during light and dark cycles over a period of 18 days. (D) 2way ANOVA to demonstrate the effects of IRW (low and high dose) on circadian rhythm in MAP. Data represented as mean ± SEM from n=6 animals per treatment group.

All data presented are mean ± SEM of 3-6 animals from each treatment group. For BP data, one –way ANOVA was used to determine the effect of IRW (Figure *1*) and a two-way ANOVA was used to determine the interaction between two factors (IRW dose and circadian rhythm of BP, Figure *2*), with a Bonferroni's post-test to compare among groups. MCh curves were fitted using nonlinear regression, and E_max_ values were compared using one-way ANOVA followed by Bonferroni's post-test or unpaired t-test as appropriate. ICAM-1, VCAM-1 and eNOS bands, plasma levels of Ang II and bradykinin as well as quantifications for nitrotyrosine and type I collagen immunostaining were analysed by unpaired *t*-tests. Data from endothelial cell experiments were analyzed by one way ANOVA with Tukey’s post test. A *p* value < 0.05 was considered statistically significant.

## Results

### IRW treatment attenuates BP in SHRs

The SHRs demonstrated already established hypertension at the beginning of the study (day 0). IRW treatment caused attenuation of BP as early as day 3 (Figure *1A*). After 18 days of IRW treatment, SBP was significantly decreased in both low and high dose groups to 191.4±2.0 mmHg and 172.1±4.7 mmHg respectively, compared to the untreated group (Figure *1A*). Similar effects were observed in MAP and DBP with high dose IRW treatment (Figure *1B and C*). Although the same trends were observed in the low dose group, they failed to reach significance. Despite the changes in BP, no changes were observed in HR (Figure *1D*).

### IRW treatment restores circadian rhythm of BP

Circadian variations or nocturnal dipping in blood pressure (MAP, SBP and DBP) were measured during the treatment period. The mean BP during each 12 h light cycle (light/dark) was calculated. The circadian variation of BP was disturbed in the untreated animals, there being little difference in blood pressure during the light and dark cycles. After treatment with both low and high doses of IRW, the impaired circadian variation in MAP, SBP and DBP were restored. Indeed, the animals in both low and high dose IRW treatment groups had the circadian variations in MAP, SBP and DBP restored (Figure *2A, B, C and D*). 

### IRW restores NO sensitive vasorelaxation

**Figure 3:**
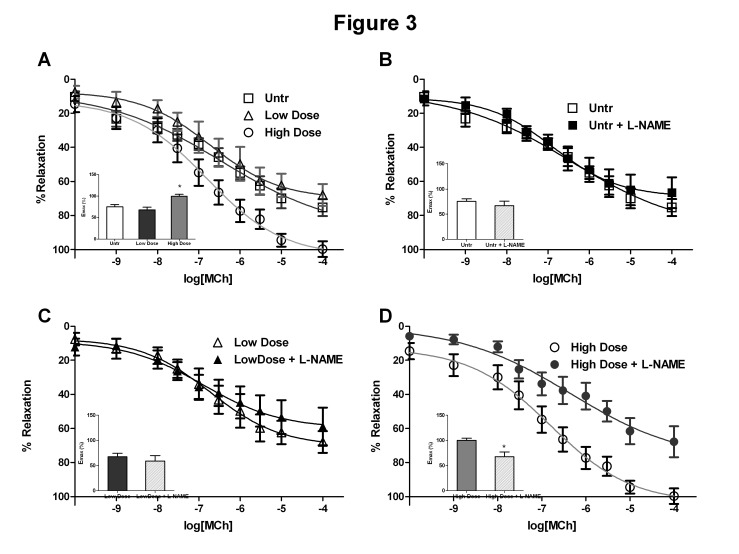
IRW treatment restores the nitric oxide contribution to vasodilatation in mesenteric arteries of SHRs. (A) IRW at the high dose (15mg/Kg BW) but not at the low dose (3mg/Kg BW) significantly increased maximal vasorelaxation in response to MCh. (B, C and D) Addition of L-NAME (100 µM) prior to MCh treatment attenuated vasorelaxation in the high dose (D) but not in the low dose (C) or the untreated (B) groups. Data represented as mean ± SEM from n=6 animals per treatment group. * indicates P<0.05 compared to the untreated group.

Vascular responses to phenylephrine (PE) constriction in the mesenteric arteries were unaffected in the IRW treatment groups compared to the untreated SHRs (*Data not shown*). As illustrated in Figure *3A*, vasodilation to MCh was significantly enhanced at high dose IRW. MCh mediated vasodilation is multifactorial, involving multiple vascular pathways such as NO, prostaglandins and endothelial derived hyperpolarizing factor (EDHF) [35]. While incubation with the NOS inhibitor L-NAME did not alter vasodilation in the untreated and low dose IRW groups, vasorelaxation in the high dose animals was significantly decreased (Figure *3B, C and D*), suggesting restoration of NO dependent vasorelaxation after IRW treatment. Given that clearly defined changes were only observed in the high dose group, all further experiments were performed only in the untreated and high dose IRW treated groups. 

### IRW appears to inhibit ACE-I in vivo

**Figure 4:**
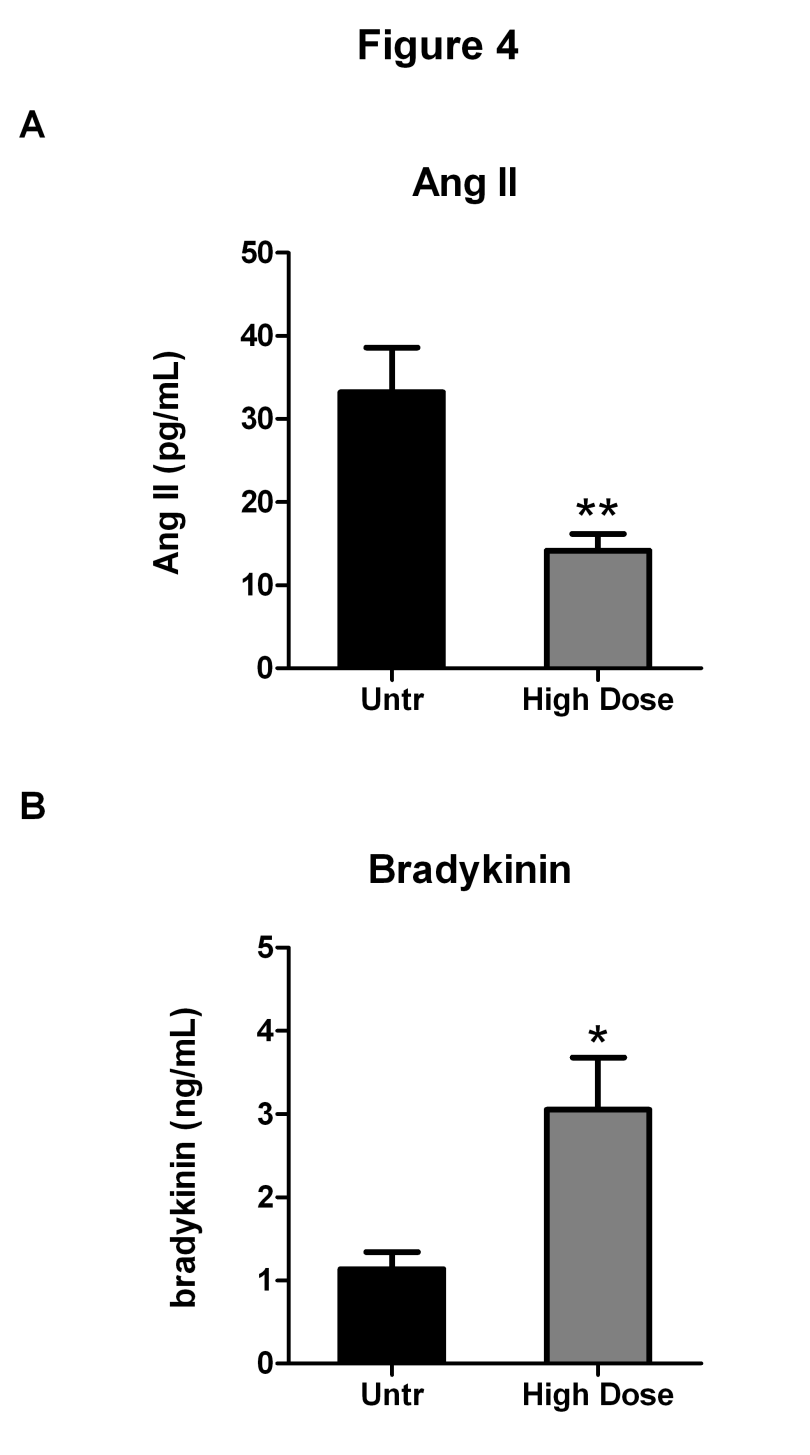
IRW treatment attenuates plasma Ang II levels through possible ACE inhibitory effects. (A) Plasma Ang II (pg/mL) levels from untreated and high dose (15mg/Kg BW) IRW treated SHRs are shown. (B) Plasma bradykinin (ng/mL) levels from untreated and high dose (15mg/Kg BW) IRW treated SHRs. Data represented as mean ± SEM from n=6 animals per treatment group. * and ** indicate P<0.05 and P<0.01 respectively, as compared to the untreated group.

The effect of high dose IRW on the SBP, MAP and DBP of SHR was associated with concomitant changes in circulating levels of Ang II and bradykinin. IRW treatment decreased Ang II levels from 35.3±5.4 pg/mL in the untreated group to 14.2±2.1 pg/mL in the high dose treated group (Figure *4A*). The treatment also increased the circulating levels of bradykinin (a molecule metabolized by ACE) which increased from 1.5±0.2 ng/mL in the untreated group to 3.0±0.6 ng/mL in the high dose treated group (Figure *4B*). These results suggest that IRW can act as an ACE inhibitor and thus decrease the production of Ang II as well as inhibit the degradation of bradykinin

### IRW ameliorates inflammation

**Figure 5:**
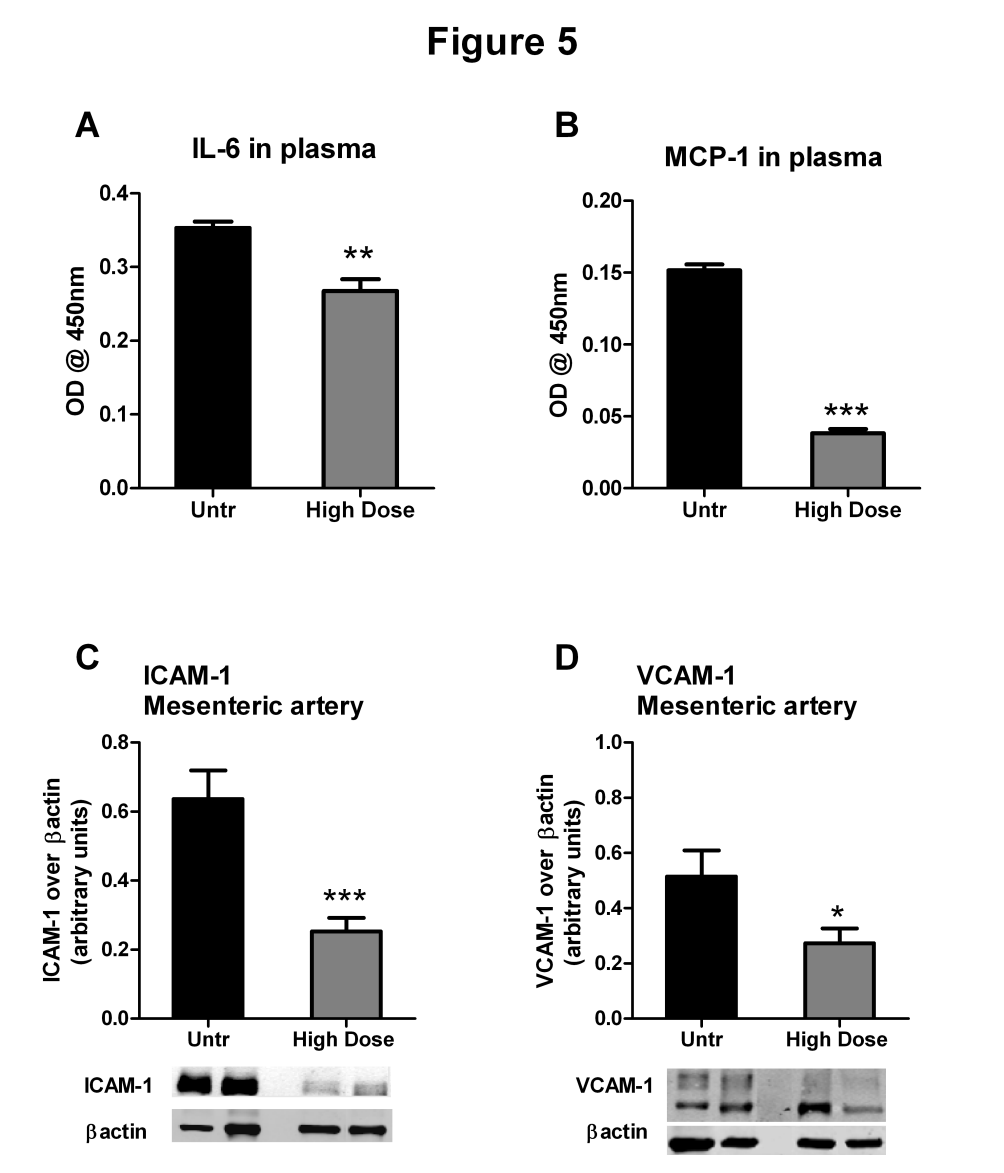
IRW treatment attenuates inflammatory markers in SHRs. (A and B) Relative changes in plasma IL-6 and MCP-1 levels in untreated and high dose (15mg/Kg BW) IRW treated SHRs. (C and D) ICAM-1 and VCAM-1 expression, normalized to ß actin in mesenteric artery lysates from untreated and high dose (15mg/Kg BW) IRW treated animals. Data represented as mean ± SEM from n= 4-6 animals per treatment group. *, ** and *** indicate P<0.05, P<0.01 and P<0.001 respectively, as compared to the untreated group.

High dose IRW treatment significantly decreased the expression of inflammatory biomarkers in plasma such as interleukin-6 (IL-6) and monocyte chemoattractant protein-1 (MCP-1), compared to the untreated animals (Figure *5A and B*). Similarly, high dose IRW also decreased the expression of inflammatory adhesion molecules, namely, ICAM-1 and VCAM-1 in mesenteric arteries (Figure *5C and D*). 

**Figure 6:**
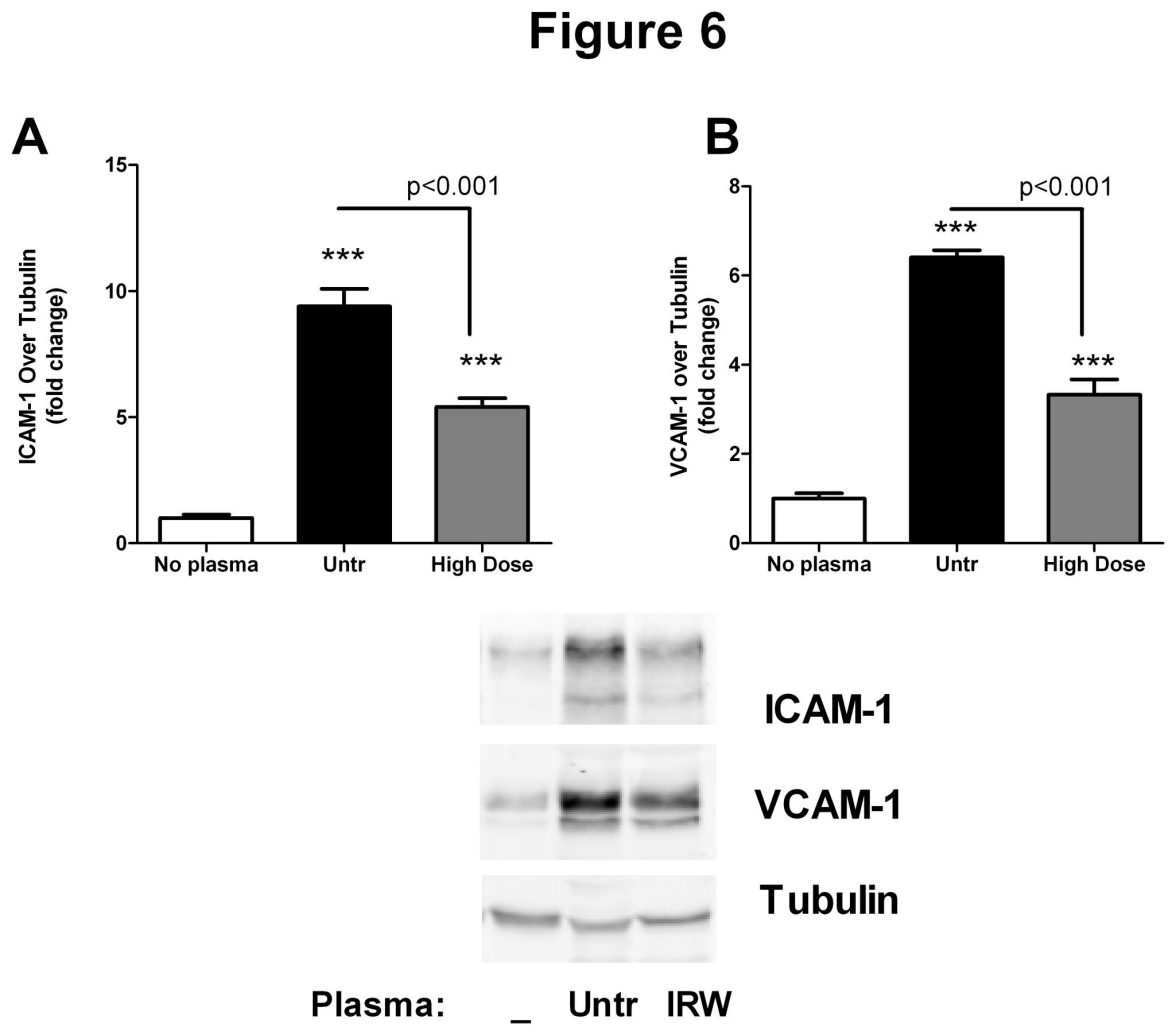
IRW treatment reduces the inflammatory potential of SHR plasma. (A and B) Confluent HUVEC monolayers were treated with 10% plasma from untreated or high dose IRW treated SHRs for 4 hours. Cells were lysed and immunoblotted for ICAM-1 and VCAM-1 levels. Data from 3-4 different experiments are summarized as mean ± SEM. A representative set of images are shown. *** indicates P<0.001 as compared to the No plasma group.

Plasma from both untreated and high dose IRW treated SHRs upregulated the expression of leukocyte adhesion molecules such as ICAM-1 and VCAM-1 in cultured human endothelial cells. However the effects with untreated plasma were much higher than that observed in the IRW treated group, suggesting at least a partial amelioration of the pro- inflammatory phenotype (Figure *6A and B*). 

### IRW restores vascular eNOS expression

**Figure 7:**
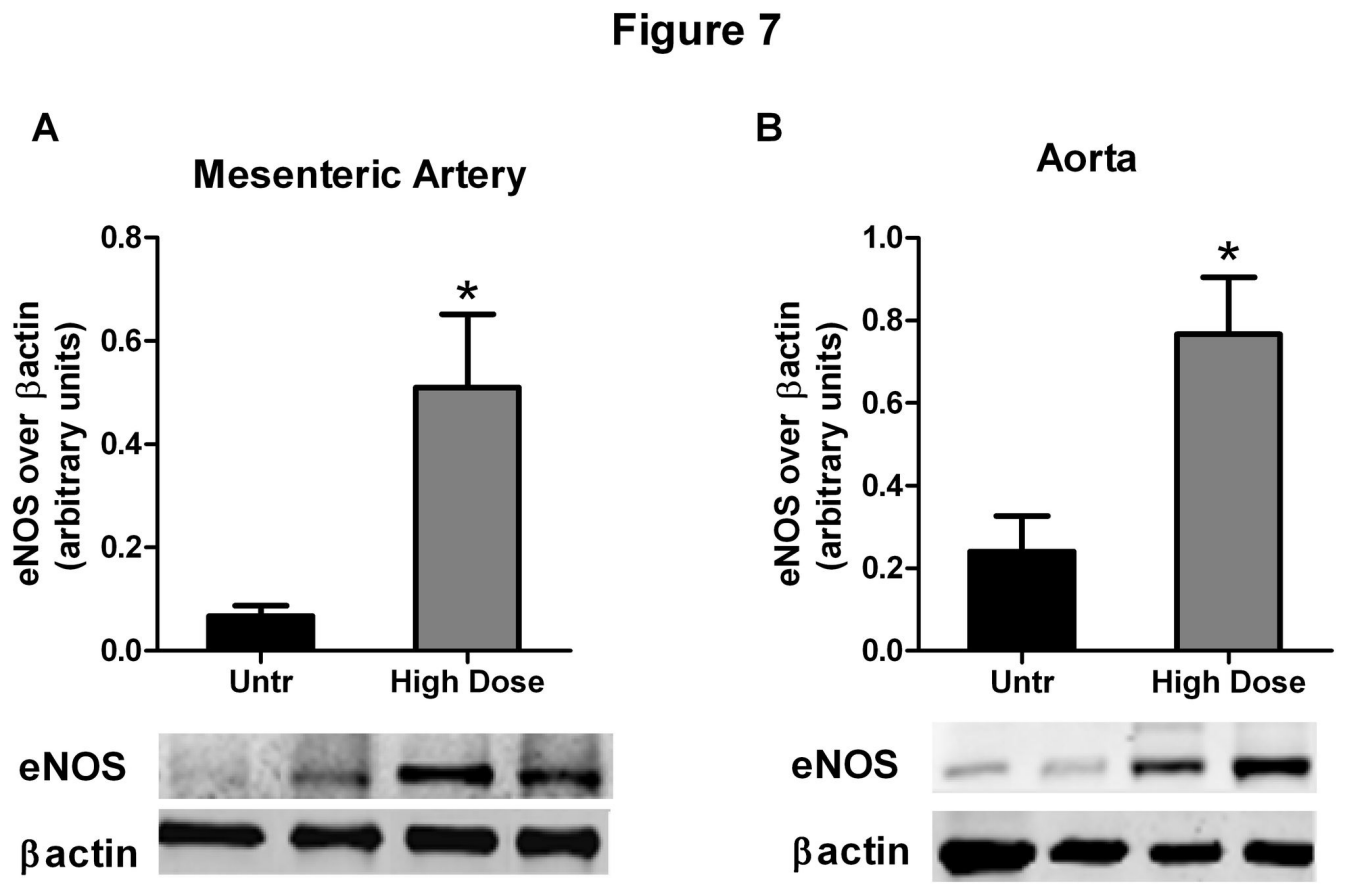
IRW treatment restores eNOS expression in SHR vasculature. Expression of eNOS, normalized to ß actin in mesenteric artery (A) and aortic (B) lysates from untreated and high dose (15mg/Kg BW) IRW treated animals. Data represented as mean ± SEM from n=6 animals per treatment group. * indicates P<0.05 compared to the untreated group.

High dose IRW treatment significantly increased the expression of eNOS in the mesenteric arteries (Figure *7A*), compared to the untreated animals. Similar effects were also observed in aortic lysates (Figure *7B*). These results suggest that IRW enhanced the expression of eNOS in the vasculature, which could potentially explain the increase in NO-mediated vasorelaxation 

### IRW ameliorates oxidative/nitrosative stress and fibrosis in vivo

**Figure 8:**
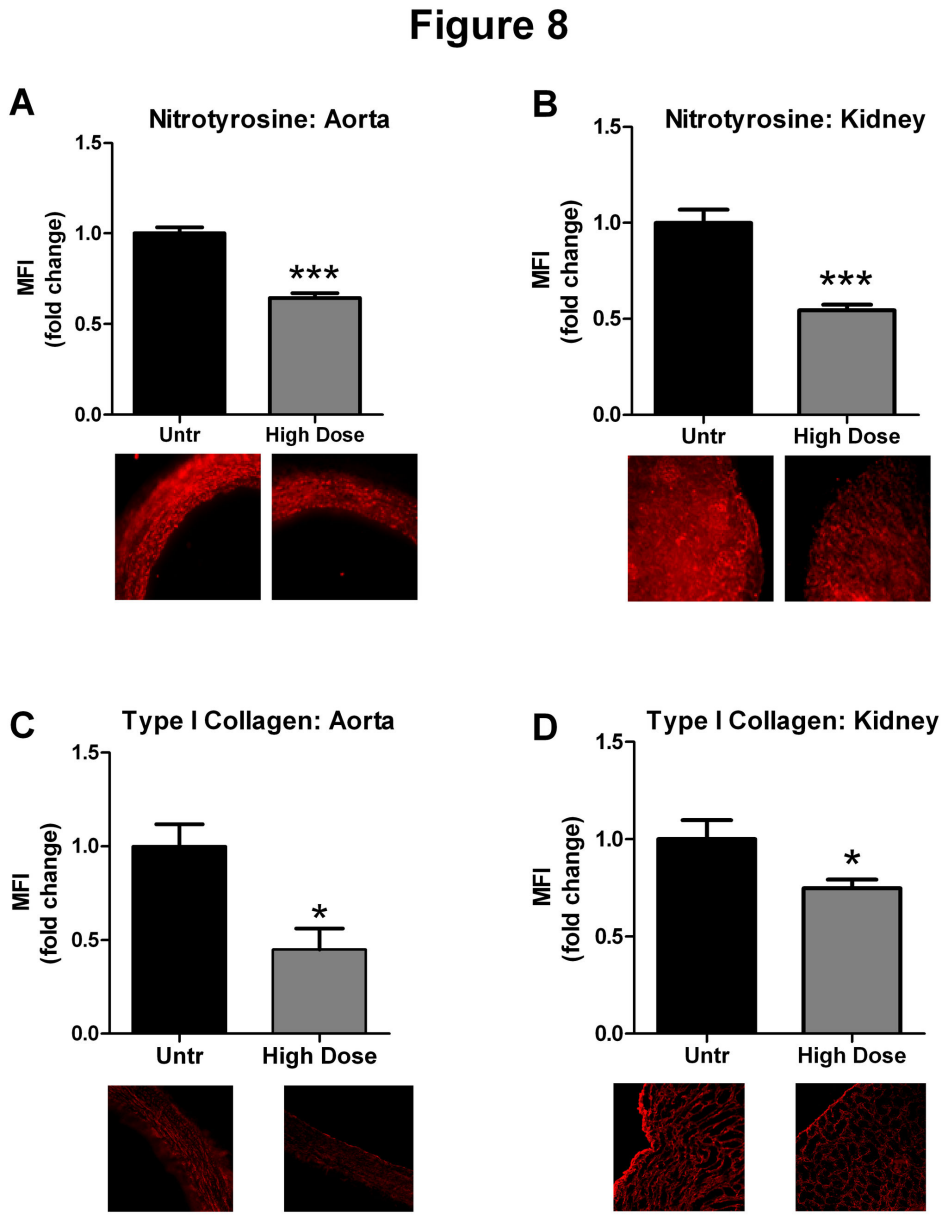
IRW treatment attenuates tissue nitrotyrosine and fibrosis in SHRs. (A and B) Immunostaining for nitrotyrosine in aortic and kidney sections from untreated and high dose (15mg/Kg BW) IRW treated SHRs. (C and D) Immunostaining for type I collagen in aortic and kidney sections from untreated and high dose (15mg/Kg BW) IRW treated SHRs. Data represented as mean ± SEM from n= 3-4 animals per treatment group. * and *** indicate P<0.05 and P<0.001 respectively, as compared to the untreated group.

High dose IRW treatment reduced oxidative/nitrosative stress in SHRs as demonstrated by a significant decrease in nitrotyrosine staining in aortas and kidneys of IRW treated animals (Figure *8A and B*). In addition, IRW treatment also reduced immunostaining for type I collagen in both aortas and kidneys, suggesting decreased tissue fibrosis (Figure *8C and D*).

## Discussion

The major findings of this study are: (i) Egg derived bioactive tri-peptide IRW significantly attenuated established hypertension in adult male SHRs, (ii) IRW treatment increased eNOS expression and increased NO-mediated vasorelaxation, (iii) IRW treatment attenuated plasma Ang II levels and ameliorated markers of inflammation, oxidative/nitrosative stress and fibrosis in SHR animals.

Data from this study may provide a natural health option for treating hypertensive complications leading to CVDs. Occurrence of CVDs is often linked to diet. This has led to an increased interest in using bioactive foods as a strategy to reduce the risk of CVDs. Thus, several active compounds have been identified in the past two decades from different food components and with various cardio-protective benefits [13,36,37]. Specifically, food derived peptides with blood pressure lowering (ACE inhibitory), cholesterol lowering, anti-thrombotic and anti-oxidant activities have been proven beneficial against CVDs [13,38,39]. Moreover, some of these peptides exhibit multiple bioactive functionalities [15]. Therefore food derived bioactive peptides, irrespective of their plant or animal origin, may exert regulatory functions on human health beyond their nutritional value. In addition to being an economically and nutritionally important food commodity, egg is a well-known source of many bioactive peptides [36,40-42]. IRW, a positively charged bioactive tri-peptide with ACE inhibitory activity, was previously identified and characterized from egg white protein ovotransferrin [17]. IRW also exhibits anti-inflammatory and anti-oxidant properties in cultured endothelial cells [18,19]. Hence the next logical step was to test the *in vivo* efficacy of IRW in an animal model of hypertension. 

SHR is a well-established model of human essential hypertension. Various studies performed to determine the anti-hypertensive effects of food derived bioactive peptides have used SHR animals as a model system [20,26]. The development of high blood pressure in these animals is similar to that in human subjects. Hypertension appears at 12-14 weeks of age in SHRs and they develop established hypertension by early adulthood [21]. Along with elevated BP, studies have shown that SHR animals have blunted diurnal (light-dark cycle) variations of BP compared to normotensive rats [43,44]. The underlying pathological mechanisms include increased activity of the renin-angiotensin system (RAS) as well as increases in vascular inflammation and oxidative stress [22,24,25]. Hence SHR is a suitable model to study the *in vivo* efficacy of IRW.

While IRW treatment significantly reduced the elevated BP in SHRs, no significant change was observed in HR, suggesting that IRW treatment did not affect the cardiac functions in this study. This is beneficial from a therapeutic point of view since the preservation of normal cardiac responses would presumably minimize the risk of side-effects such arrhythmias and related complications. IRW also restored the attenuated circadian variation in BP which is characteristically observed in SHR. Given that loss of “nocturnal dipping” can contribute to clinical events like myocardial ischemia, acute myocardial infarct and sudden cardiac death [45-47], the restoration of impaired nocturnal dipping in blood pressure by IRW may help to prevent target organ damage in hypertension.

IRW appears to act through multiple pathways leading to lowered BP in SHRs. It is known that loss of vascular eNOS activity causes endothelial dysfunction and contributes to the pathogenesis of hypertension and atherosclerosis [48,49]. In our study, no significant difference in vascular relaxation has been observed after L-NAME treatment in the mesenteric arties of untreated SHRs, suggesting that SHRs may have impaired NO dependent vasorelaxation. Indeed, a study by Bagnost et al. has demonstrated the loss of NO dependent vasorelaxation in SHRs compare to their wild type (WKY) controls [50]. We also found that IRW upregulated eNOS and enhanced NO mediated vasorelaxation in SHRs, suggesting the rectification of endothelial dysfunction as seen in the untreated animals. Based on our previous study we postulated that IRW would also exhibit ACE inhibitory activity *in vivo* [17]. Indeed IRW treatment reduced the plasma Ang II levels with a corresponding increase in bradykinin, suggesting an ACE inhibitory effect. 

We have previously demonstrated that IRW reduces TNF-induced upregulation of MCP-1, ICAM-1 and VCAM-1 in an endothelial cell culture system [18,19]. A similar effect was observed *in vivo* in the current study; IRW decreased levels of the inflammatory cytokines/chemokines IL-6 and MCP-1. This phenomenon was accompanied by decreases in expression of inflammatory adhesion molecules (ICAM-1 and VCAM-1) in vascular tissues, suggesting generalized anti-inflammatory effects of IRW *in vivo*. In addition, plasma from IRW treated SHRs induced lower levels of ICAM-1 and VCAM-1 expression in cultured endothelial cells, further supporting a reduction in proinflammatory properties. Given that SHRs have increased circulating levels of pro-inflammatory cytokines which may contribute to the endothelial dysfunction and upregulate leukocyte adhesion molecules in the vasculature, we propose that controlling the inflammatory pathways with IRW could potentially ameliorate the vascular pathologies. 

Increased oxidative stress contributes to the pathology of hypertension [51]. Reactive oxygen species (ROS) such as superoxide can interact with NO to generate peroxynitrite (-ONOO), a highly reactive species that contributes to oxidative /nitrosative stress [52]. Peroxynitrite leads to tyrosine nitration of various proteins and contributes to a pro-inflammatory phenotype. Studies have demonstrated that reductions in ROS and –ONOO levels in SHRs can reduce blood pressure, suggesting a role for these species on the pathology [53,54]. Our study showed that high dose IRW could reduce nitrotyrosine levels in both aorta and kidneys, suggesting an anti-oxidant effect of IRW on these tissues. These findings are in accordance with our previous study showing anti-oxidant effects of IRW on the human endothelium [19]. Inflammatory and oxidative processes can also lead to increased fibrosis and consequent remodeling in various tissues. SHR animals are prone to fibrotic changes in renal and aortic tissues, which may further contribute to the complications of hypertension [55]. IRW treatment significantly attenuated type I collagen levels both in kidney and aorta, suggesting a reduction in hypertension induced tissue remodeling.

The biological activity of orally administered peptides is highly dependent on their ability to reach the target site without being degraded and/or inactivated by intestinal or plasma peptidases. A previous study on milk derived bioactive tripeptides has shown evidence for oral absorption without degradation [56]. Our previous study in cells has shown the importance of integrity of IRW in exerting anti-inflammatory and antioxidant activities [19] ; the current study has now conclusively demonstrated the *in vivo* efficacy of IRW, further supporting its oral bioavailability. This finding on oral bioavailability of IRW is in accordance with our previous findings using an intestinal epithelial cell culture system where the peptide was observed to cross the epithelial barrier [57], although metabolism of IRW *in vivo* has not been studied yet. 

In this study, we used only male animals and had a limited study period of 18 days. Future studies may incorporate animals of both sexes, a longer treatment period and observation of the effects of treatment withdrawal on BP regulation to avoid the limitations of the present study. Future studies could also involve elucidation of the molecular mechanisms underlying the anti-inflammatory and NO generating effects observed *in vivo*. 

In summary, the *in vivo* anti-hypertensive effects of orally given IRW appear to be mediated through several different pathways, such as, increased NO mediated vasodilation, regulating RAS through ACE inhibition and reducing vascular inflammation. Additionally, IRW could restore the disturbed circadian variations of blood pressure in these animals. These findings might support the use of egg derived peptide as a functional food or nutraceutical ingredient with potential applications in the prevention and management of hypertension.
